# Supplementary material for: Local-scale Arctic tundra heterogeneity affects regional-scale carbon dynamics
Source: Nat Commun. 2020 Oct 1;11:4925. doi: 10.1038/s41467-020-18768-z (PMC7529807; doi:10.1038/s41467-020-18768-z)
Supplement: Supplementary file 1 — Supplementary Information [file 41467_2020_18768_MOESM1_ESM.pdf]

Supplementary Information for:

Local-scale Arctic tundra heterogeneity affects regional-scale carbon dynamics

M.J. Lara et al.

Supplementary Table 1: Observations used to benchmark DOS-TEM simulations from all available data sources. We recalculated benchmarks for landform groups by weighting data by the prevalence of each landform. GPP=Gross Primary Productivity (gC m^{-2} yr^{-1}), NPP= Net Ecosystem Productivity (gC m^{-2} yr^{-1}), Veg C= Vegetation Carbon (gC m^{-2}), Veg N= Vegetation Nitrogen (gN m^{-2}), Avl N = Available Nitrogen (gN m^{-2}), Tot Soil N = Total Soil Nitrogen (gN m^{-2}), Tot Soil C= Total Soil Carbon (gC m^{-2}), Fib= Fibric layer thickness (m), Hum= Humic layer thickness (m), CFALL= Vegetation Carbon lost as litter(gC m^{-2} yr^{-1}), Fib C= Fibric layer soil carbon content (gC m^{-2}), Hum C= Humic layer soil carbon content (gC m^{-2}), and Min C= Mineral layer soil carbon content (gC m^{-2}).

Cluster	Landform	GPP*	NPP	Veg C	Veg N	Avl N	Tot Soil N	Tot Soil C	Clay	Sand	Silt	Fib	Hum	CFALL*	Fib C	Hum C	Min C
6	DS	119	79	256	6.2	3.6	4079	80952	34	32	32	0.05	0.54	0.053	4048	43714	33190
	HC	128	85	286	6.2	3.6	3357	66650	51	13	35	0.1	0.22	0.078	6665	14663	45322
	FC	314	209	502	10.9	1.4	2685	53314	42	29	29	0.11	0.02	0.079	5864	1066	46383
	LC	296	197	524	11.1	0.5	3121	61957	51	16	33	0.17	0.14	0.082	10533	8674	42751
	nDTLB	420	280	640	13.3	1	2587	51377	63	20	17	0.13	0.19	0.08	6679	9762	34936
	Pond	342	228	299	7.5	1.1	3329	66080	30	5	65	0.26	0.56	0.081	17181	37005	11894
5	DS	119	79	256	6.2	3.6	4079	80952	34	32	32	0.05	0.54	0.053	4048	43714	33190
	HC	128	85	286	6.2	3.6	3357	66650	51	13	35	0.1	0.22	0.078	6665	14663	45322
	FC+LC	303	202	515	11	0.9	2941	58391	47	21	31	0.15	0.09	0.081	8607	5535	44249
	nDTLB	420	280	640	13.3	1	2587	51377	63	20	17	0.13	0.19	0.08	6679	9762	34936
	Pond	342	228	299	7.5	1.1	3329	66080	30	5	65	0.26	0.56	0.081	17181	37005	11894
4	DS	119	79	256	6.2	3.6	4079	80952	34	32	32	0.05	0.54	0.053	4048	43714	33190
	HC	128	85	286	6.2	3.6	3357	66650	51	13	35	0.1	0.22	0.078	6665	14663	45322
	FC+LC+Mdw	309	206	521	11.2	0.9	2923	58035	48	21	31	0.14	0.1	0.081	8384	5545	44105
	Pond	342	228	299	7.5	1.1	3329	66080	30	5	65	0.26	0.56	0.081	17181	37005	11894
3	DS	119	79	256	6.2	3.6	4079	80952	34	32	32	0.05	0.54	0.053	4048	43714	33190
	HC	128	85	286	6.2	3.6	3357	66650	51	13	35	0.1	0.22	0.078	6665	14663	45322
	FC+LC+Mdw+ nDTLB+ Pond	311	207	506	10.9	0.9	2950	58566	47	20	33	0.15	0.13	0.081	8908	7392	42266
2	DS+HC	124	83	274	6.2	3.6	3638	72223	44	20	34	0.08	0.34	0.068	5815	24894	41513
	FC+LC+Mdw+ nDTLB+ Pond	311	207	506	10.9	0.9	2950	58566	47	20	33	0.15	0.13	0.081	8908	7392	42266
1	All	240	160	410	8.9	1.6	2930	58168	43	19	31	0.12	0.18	0.072	7138	10205	40825

**Computed following Euskirchen et al.* [1]

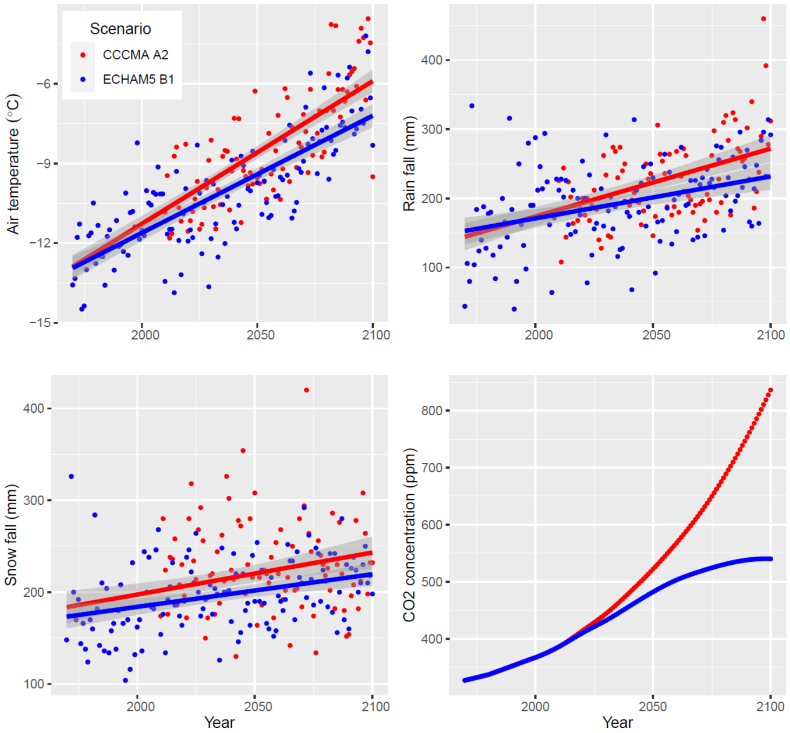

Supplemental Figure 1: Historical and projected air temperature (top left), rain fall (top right), snow fall (bottom left), and atmospheric carbon dioxide concentrations (bottom right) used for driving model simulations between 1970-2100. Climate model and emission scenarios CCCMA A2 and ECHAM5 B1 are displayed in red and blue colors, respectively.

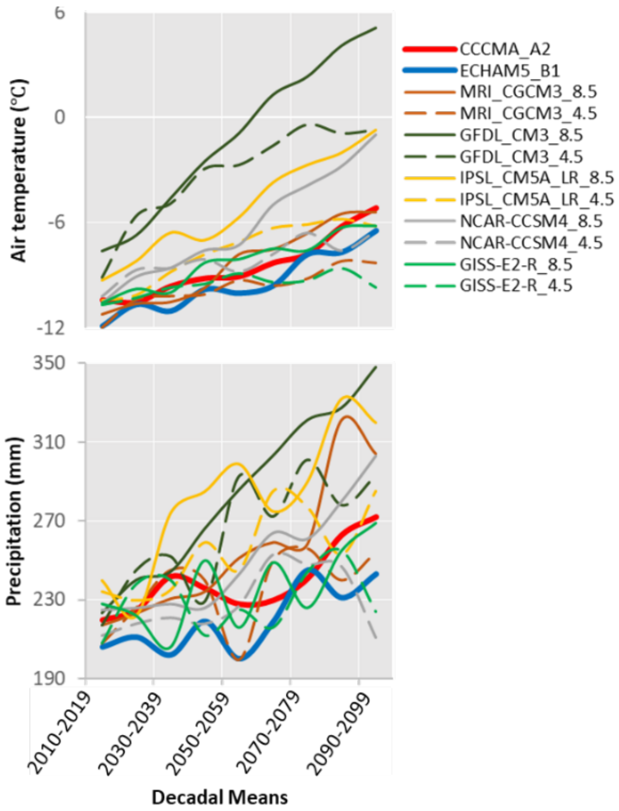

Supplemental Figure 2: Comparison of projected decadal mean temperature and precipitation among climate and emission scenarios. Scenarios include CCCMA A2 (red line), ECHAM5 B1 (blue line), and the five best-performing CMIP5 climate models (RCP 8.5 and 4.5) for the Arctic[2]. CMIP5 climate models include the Meteorological Research Institute Coupled General Circulation Model v3.0 (MRI-CGCM3), NOAA Geophysical Fluid Dynamics Laboratory Coupled Model 3.0 (GFDL-CM3), Institut Pierre-Simon Laplace Coupled Model v5A (IPSL-CM5A-LR), National Center for Atmospheric Research Community Earth System Model 4 (NCAR-CCSM4), and the NASA Goddard Institute for Space Studies ModelE/Russel (GISS-E2-R).

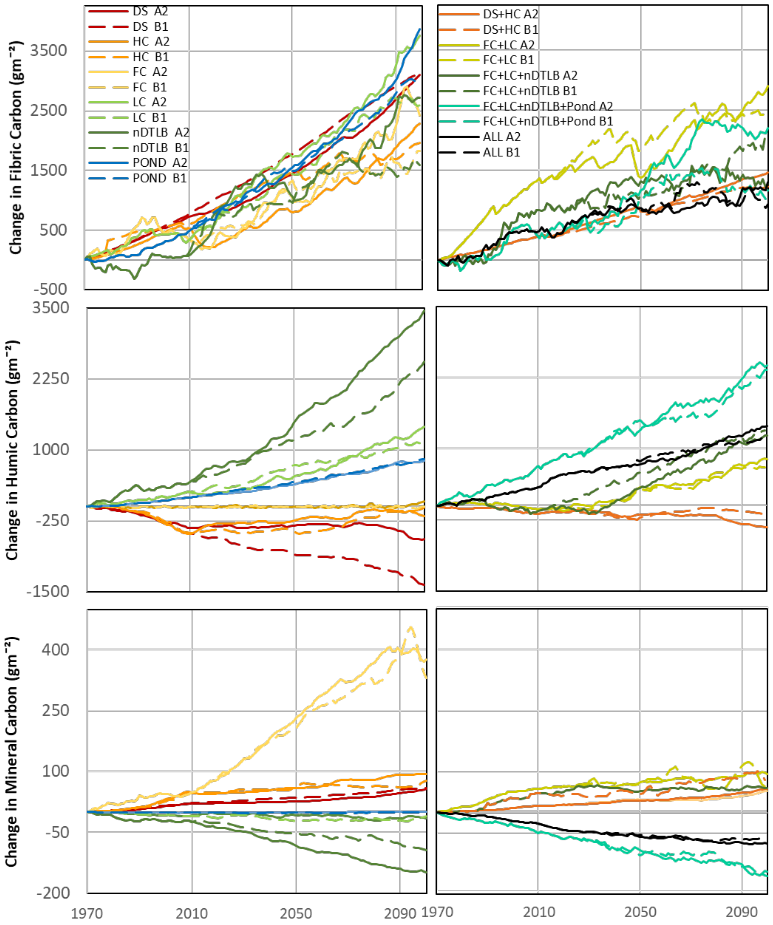

Supplementary Figure 3: Simulated change in soil carbon through 2100. Simulations display all polygonal tundra landforms, landform groups, and soil horizons, forced using high (CCCMA A2) and low (ECHAM5 B1) climate and emission pathways for the Barrow Peninsula. Climate forcing for CCCMA A2 and ECHAM5 B1 are similar to that produced by RCPs 6.0 and 4.5.

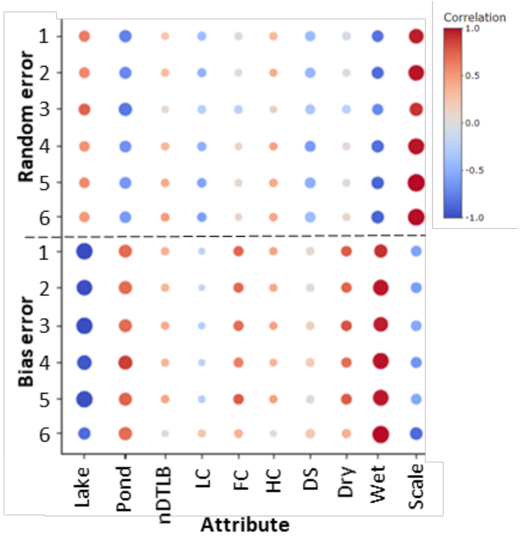

Supplementary Figure 4: Pearson's correlations of model uncertainty metrics random error and bias error by cluster and spatial attributes. The larger the bubble the greater p-value. Landform categories Dry and Wet include spatial data from "DS+HC" and "FC+LC+nDTLB+Pond", respectively.

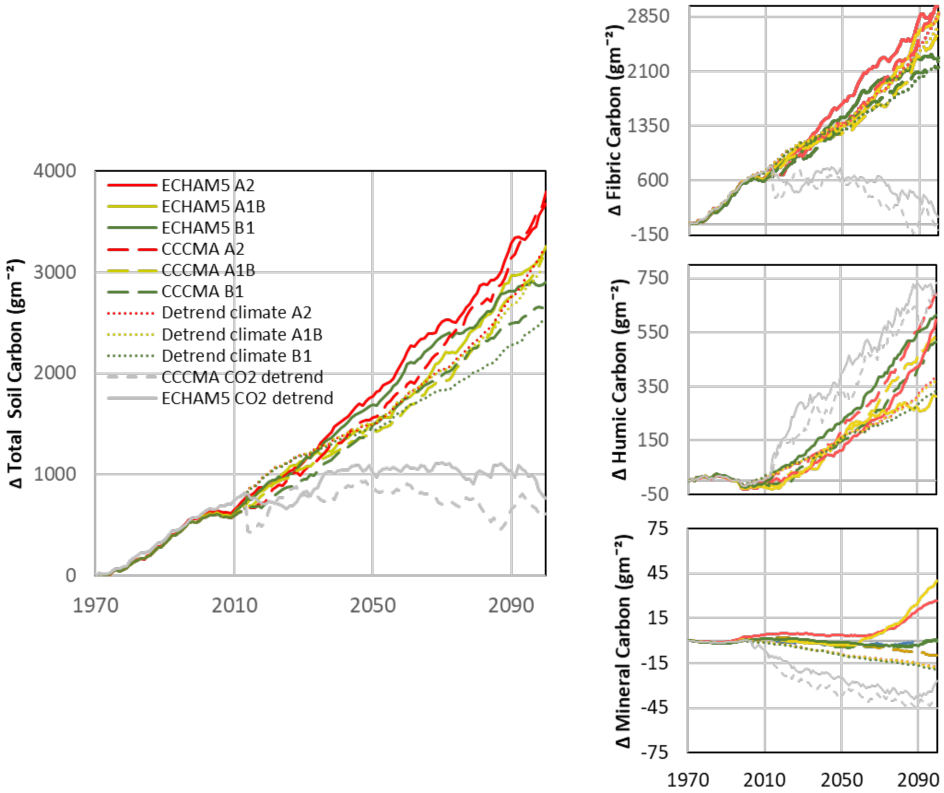

Supplementary Figure 5: Cumulative change in total soil carbon in response to climate and CO_2 forcing on the Barrow Peninsula. Ensemble simulations are summed over all soil horizons (left panel) and individual soil horizons (right column). Detrended climate simulations are represented by dotted lines and detrended atmospheric CO_2 simulations are represented by grey lines.

Supplementary References

1. Euskirchen, E. S., Mcguire, A. D., Chapin, F. S., Yi, S. & Thompson, C. C. Changes in vegetation in northern Alaska under scenarios of climate change, 2003-2100: implications for climate feedbacks. *Ecol. Appl.* (2009) doi:10.1890/08-0806.1.
2. Walsh, J. E. *et al.* Downscaling of climate model output for Alaskan stakeholders. *Environ. Model. Softw.* (2018) doi:10.1016/j.envsoft.2018.03.021.
